# MicroRNAs in renal cell carcinoma: A systematic review of clinical implications (Review)

**DOI:** 10.3892/or.2015.3799

**Published:** 2015-02-13

**Authors:** MING LI, YING WANG, YONGSHENG SONG, RENGE BU, BO YIN, XIANG FEI, QIZHEN GUO, BIN WU

**Affiliations:** 1 Department of Urology, Shengjing Hospital of China Medical University, Shenyang, Liaoning 110004, P.R. China; 2 Department of Cell Biology, Harvard Medical School, Boston, MA 02115, USA; 3 Department of Nuclear Medicine, The First Affiliated Hospital of China Medical University, Shenyang, Liaoning 110001, P.R. China

**Keywords:** renal cell carcinoma, microRNA, diagnosis, prognosis, therapy

## Abstract

Despite recent advances in the understanding of the biology of renal cell carcinoma (RCC), successful surgical treatment and implementation of novel-targeted therapies, the prognosis for RCC patients remains poor. Late presentation, tumor heterogeneity and in particular the lack of molecular biomarkers for early detection, classification and the surveillance of RCC treatments are major obstacles. The increasing knowledge regarding the functional role of microRNAs (miRNAs) in pathophysiological processes may provide an important link to the identification of suitable therapeutic targets and diagnostic/prognostic biomarkers for RCC. The aim of this review was to provide new insight into the function of miRNAs in the pathogenesis of RCC and to emphasize their potential as diagnostic and prognostic markers, as well as therapeutic targets.

## 1. Introduction

Renal cell carcinoma (RCC) is the most common malignant solid tumor in adults. A total of 63,920 new cancer cases and 13,860 deaths from kidney and renal pelvis cancer were estimated to occur in the United States in 2014 (1). There are four major histologic subtypes of RCC, the clear cell RCC (ccRCC) comprise the main histological category that accounts for 75% of cases, followed by the papillary RCC (pRCC) (12%), the chromophobic RCC (chRCC) and oncocytomas (4% each) and rare subtypes (5%) (2). Although surgical resection remains the best curative therapy approach for RCC, ~20–30% of these patients experience local and/or distant disease recurrence (3). Moreover, up to 30% of patients have metastases at the time of the initial diagnosis (4). However, RCC has a highly resistant phenotype to conventional therapeutic modalities, including chemotherapy and radiation, thus it remains an extremely lethal disease (5,6). Another issue concerning RCC is the absence of biomarkers for the early detection and follow-up of the disease, which complicates the early diagnosis and makes it a challenge for the field of oncology (7). Additionally, besides the clinicopathological parameters, there are no molecular markers for the prognosis of RCC.

MicroRNAs (miRNAs) are a class of ~22 nucleotide non-coding RNA molecules that negatively regulate the expression of a wide variety of genes mainly through direct interaction with the 3′-untranslated regions (3′-UTR) of corresponding mRNA targets (8). Since being identified in 1993 by Lee *et al* (9), miRNAs have unraveled new mechanisms for the regulation of gene expression and have provided new directions for cancer research. miRNAs are pivotal regulators of all hallmarks of cancer, which include cell growth and cell cycle control, evasion of apoptosis, tissue invasion and metastasis, angiogenesis and unlimited replicative potential (10). Investigations conducted on miRNAs in RCC have increased at an exponential rate. In the present review, we systematically describe the profiling of miRNAs in RCC and their roles in renal carcinogenesis, diagnosis, prognosis and the potential roles in RCC therapy.

## 2. Biogenesis of microRNA

**Figure 1:**
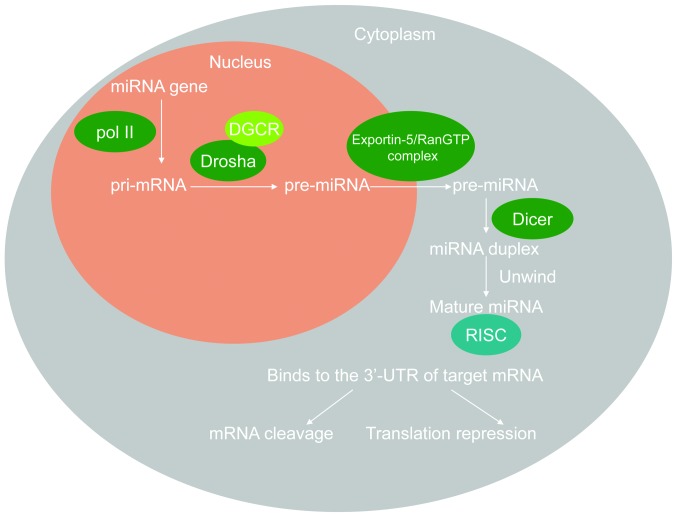
microRNA biogenesis and processing mechanism.

Most miRNAs are produced from either intergenic or intronic regions of coding or non-coding genes (11,12). They are transcribed primarily by RNA polymerase II (pol II) as part of longer primary miRNA (pri-miRNA) transcripts that are capped, spliced, and polyadenylated (13,14). The first step in pri-miRNA maturation is carried out in the nucleus by the RNase III enzyme Drosha and its cofactor DGCR8. This step produces the precursor miRNA hairpin (pre-miRNA) (15,16). The pre-miRNA was then exported to the cytoplasm by the Exportin-5/RanGTP complex where it is cleaved by Dicer to generate the double-trand miRNA (17,18). A helicase then unwinds the duplex into mature miRNAs (19). Mature miRNAs are incorporated into the RNA-induced silencing complex (RISC) and bind to the complementary 3′-UTR of their specific target mRNAs. This process results in the inhibiton of mRNA translation or promotes its degradation and leads to post-transcriptional gene silencing (20–22) (Fig. 1).

## 3. microRNAs in renal cell carcinoma

A number of approaches have been developed to quantify miRNA levels and numerous studies on miRNA expression profiles and the determination of their mRNA targets and the functional analysis have been carried out in RCC. The deregulated miRNAs in RCC are presented in Tables I and II (23–64). The microarray-based experiments identified 13 overexpressed and 20 downregulated miRNAs in RCC samples. Expression in ccRCC tissue samples compared with matched non-malignant samples measured by RT-PCR was increased on average by 2.7- to 23-fold for the miR-16, -452^*^, -224, -155 and -210, but decreased by 4.8- to 138-fold for miR-200b, -363, -429, -200c, -514 and -141 (65). Gottardo *et al,* using miRNA microarray hybridization analysis found that miR-28, -185, -27, and let-7f-2 were significantly upregulated in RCC compared to normal kidney (66). Results of another study showed that miR-34a, -224 and -21 were upregulated, whereas miR-141, -149 and -429 were downregulated in the ccRCC tissues (67). Faragalla *et al* also found the expression of miR-21 was significantly upregulated in RCC compared with healthy kidney. A significant difference was found in the expression levels between RCC subtypes, with the highest levels of expression in ccRCC and pRCC subtypes. Significantly higher miR-21 levels were associated with higher stage and grade (68). However, Silva-Santos *et al* reported that RCC exhibited significantly lower expression levels of miR-21, -141 and -200b compared with that of normal tissues, and expression levels of all miRNAs differed significantly between malignant and benign renal cell tumors (69).

Recent findings have shown that miR-10b/-19a/-19b/-20a/ -29a/-29b/-29c/-100/-101/-126/-127/-130/-141/-143/-145/-148a/ -192/-194/-200c/-210/-215/-370/-514 were downregulated in metastatic tissue samples compared with normal tissue (70). In addition, a miRNA signature that distinguishes between metastatic and non-metastatic ccRCC was detected, including miR-451, -221, -30a, -10b and -29a, as well as a group of 12 miRNAs, including let-7 family, miR-30c, -26a, which were decreased in highly aggressive primary metastatic tumors (71).

Circulating and urinary miRNAs have also been found to be deregulated in RCC. Redova *et al* identified 30 miRNAs that were differentially expressed between the serum of RCC patients and healthy controls: 19 miRNAs were upregulated and 11 miRNAs were downregulated in RCC patients. Levels of miR-378 were increased, while those of miR-451 were decreased in the serum of RCC patients (72). In another study, miR-34a, -21 and -224 were upregulated, miR-141 was down-regulated in the sera of patients with ccRCC, and the serum miR-21 expression levels were significantly correlated with the clinical staging of the patients with ccRCC (67). It was found that RCC patients presented higher circulating expression levels of miR-221 and -222 than healthy individuals. The RCC patients with metastasis at diagnosis also presented higher circulating expression levels of miR-221 than patients without metastasis (73). However, whether these changes are the cause of RCC or as a consequence of RCC remain to be determined.

## 4. Pathophysiology of renal cell carcinoma

### VHL/HIF signaling pathway

RCC is frequently associated with inactivation of the von Hippel-Lindau (VHL) gene, resulting in elevated levels of hypoxia-inducible transcription factors (HIF). Increasing evidence supports the involvement of alternative mechanisms in the regulation of VHL/HIF expression, including suppression by miRNAs. For example, both the VHL and hypoxia-inducible factor 1-α (HIF1α) gene were direct targets of miR-17-5p and miR-224 in RCC (74). In addition, miR-138 targeted HIF1α and suppressed its expression, and affected the apoptosis and migration of ccRCC cells (32).

By contrast, miRNAs can also be regulated by VHL in an HIF-dependent or -independent manner in RCC (75), thereby affecting downstream signaling. miR-210 has been shown to be expressed at significantly higher levels in tumors with either VHL mutations or methylation of the VHL promoter and to be correlated with the expression of CA9, a known transcriptional target of HIF transcription factors (75). In another study, due to HIF1α accumulation, miR-210 upregulation induced aneuploidy via E2F3 downregulation at least in part, and played a role in tumorigenesis and/or progression of RCC (62). In addition, miR-31, -21, and let-7i were upregulated in RCC cells with functional VHL, whereas miR-155, -193b, -17, -18a, -20a, and -210 were downregulated. The knockdown of HIF1A or HIF1B also reduced miR-210 and miR-155 expression levels (75).

### PI3K/Akt signaling pathway

The upregulation of miR-122 was shown to play an important role in the progression of RCC by activating the PI3K/Akt signaling pathway and is a potential molecular target for anti-cancer therapeutics (76). Findings of another study showed a molecular order of a phosphatase-kinase couple involving PTEN/Akt/IKKβ and NFκB-dependent cyclin D1 expression for renal carcinoma cell proliferation by increased miR-21 levels (77). Furthermore, miR-21 was shown to directly downregulate the proapoptotic protein PDCD4 to increase the migration and invasion of ACHN and 786-O RCC cells as a result of the phosphorylation/activation of Akt and IKKβ, which activated NFκB-dependent transcription. Thus, miR-21 promoted cancer cell hyperplasia and contributed to tumor cell transformation and metastasis (78). In addition, miR-200c decreased the metastatic ability of RCC cells by upregulating E-cadherin through ZEB1. Additionally, modulation of the expression of miR-200c influenced Akt protein levels, suggesting the presence of an Akt-miR-200c-E-cadherin axis in the epithelial-to-mesenchymal transition (EMT) process in RCC (43).

### Methylation and miRNA

miRNAs have been shown to play a role as targets and effectors in gene hypermethylation and silencing in cancer cells. Two genes encoding for miR-9 were demonstrated to be significantly hypermethylated in ccRCC tumors compared with adjacent normal tissues resulting in decreased expression. Additionally, the methylation of these genes was more significant in DNA obtained from the primary tumor for patients who developed a recurrence than in tumors from non-recurrent patients. Furthermore, methylation of miR-9-3 was significantly associated with an increased risk of recurrence, and high methylation levels of miR-9-1 or -9-3 resulted in a significant, almost 30-month decrease in recurrence-free survival time (79). Another study showed that hypermethylation of miR-124-3 in samples of RCCs was identified compared with adjacent normal tissues. miR-124-3 methylation was significantly increased in tumors with state of advanced disease. Higher relative methylation was associated with worse recurrence-free survival. Moreover, miR-124-3 CpG island (CGI) methylation was identified as a relevant epigenetic marker for ccRCC, while methylation of miR-124-3 was suggested as an independent prognosticator for ccRCC (80). In addition, the frequency of methylation of miR-124a-2, -124a-3, -9-1, -9-3, -34b/c and -129-2 was significantly higher in tumor samples than in normal tissues (81). On the other hand, DNA promoter methylation levels were found to be inversely correlated with the expression of miR-21, -10b and -30a in ccRCC (82).

### Other novel mechanisms

The fundamental role of miRNAs on the pathophysiology of RCC involve the downregulation of their target genes by recognizing the 3′-UTR, through which, they act as oncogenes or tumor suppressors and affect the biology of cell processes such as proliferation, migration and invasion (Tables I and II). However, other mechanisms are also involved.

Single-nucleotide polymorphisms (SNPs) in miRNAs genes are currently being identified for contributing to cancer risk, prognosis and survival. miR-196a2 SNP rs11614913 was associated with RCC susceptibility in a recessive model and with survival of RCC in a dominant model (83).

Chromosomal instability enables tumor development, in part by aberrant expression of the mitotic checkpoint protein Mad2. In VHL-positive RCC cells, enhanced expression of miR-28-5p decreased Mad2 levels and promoted checkpoint weakness and chromosomal instability. Conversely, in checkpoint-deficient VHL-negative RCC cells, inhibition of miR-28-5p function restored Mad2 levels, mitotic checkpoint proficiency, and chromosomal stability (84).

Of note, Prior *et al* identified a novel paracrine mechanism through which high miR-942 levels in metastatic renal cell carcinoma (mRCC) cells upregulate MMP-9 and VEGF secretion to enhance endothelial migration and sunitinib resistance (85).

## 5. Biomarkers and diagnosis

Recent studies identified a number of novel deregulated miRNAs specific for each subtype of RCC and these miRNAs were able to discriminate ccRCC from the normal kidney (86,87). miR-141 was demonstrated as a potential biomarker for discriminating ccRCC from normal tissues and a crucial suppressor of ccRCC cell proliferation and metastasis by modulating the EphA2/p-FAK/p-AKT/MMPs signaling cascade (35). Another study showed that malignant and non-malignant tissue were clearly differentiated by their miRNA profile, and a combination of miR-141 and -155 resulted in a 97% overall correct classification of samples (65). Moreover, the miR-141 or -200b panel accurately distinguished RCC from normal kidney, oncocytoma from RCC and chRCC from oncocytoma (69). In addition, Faragalla *et al* demonstrated that miR-21 expression distinguished ccRCC and pRCC from chRCC and oncocytoma with 90% specificity and 83% sensitivity (68). Youssef *et al* developed a classification system that can distinguish the different RCC subtypes using unique miRNA signatures in a maximum of four steps. The system has a sensitivity of 97% in distinguishing normal from RCC, 100% for ccRCC, 97% for pRCC, and 100% accuracy in distinguishing oncocytoma from chRCC (88).

As extracellular miRNAs such as serum or urine miRNAs were also deregulated in RCC patients, they are considered promising candidates as biomarkers for the diagnosis and prognosis of RCC. The levels of miR-378 were increased, while those of miR-451 were decreased in the sera of RCC patients, and they were shown to be able to distinguish RCC from healthy controls. The combination of the two miRNAs improved stratification power with a sensitivity of 81% and a specificity of 83% and AUC =0.86 (72). Serum miR-1233 levels were increased in RCC patients with a sensitivity of 77.4% and a specificity of 37.6%. The circulating miR-1233 was identified as a potential biomarker for RCC patients (89). In addition, serum miR-210 upregulation occurred in the early stage of ccRCC (90), and serum miR-210 levels were significantly higher in ccRCC patients than in normal controls with a sensitivity of 81.0% and specificity of 79.4% in discriminating diagnosis (91). In urine, upregulated miR-15a was measured from patients with RCC but was almost undetectable in oncocytoma, other tumors, and urinary tract inflammation (92).

The high degree of diagnostic accuracy suggests that miRNA in RCC patients may serve as next-generation biomarkers for detection of the disease. However, large-scale investigations and additional improvements are required to confirm the results and verify the feasibility of routine clinical utilization (93).

## 6. Prognosis

Increasing studies showed that aberrant miRNA expression is associated with 5-year survival, overall survival, disease grade and stage, recurrence and metastasis. miRNAs with low expression associated with poor prognosis (shorter survival or early recurrence) included miR-187 (40), -215 (41), -217 (45), 155 (94) and -1826 (53). In addition, the expression levels of miR-143, -26a, -145, -10b, -195 and -126 were lower in the tumors of RCC patients who developed tumor relapse, while the lowest levels of these miRNAs were identified in primary metastatic tumors. By using Kaplan-Meier analysis, miR-127-3p, -145 and -126 were significantly correlated with relapse-free survival of non-metastatic RCC patients (95). On the other hand, a high or positive expression of miR-21 (68), -23b-3p (59), -100 (96) and -630 (97) was associated with shorter survival or early recurrence.

Metastasis is extremely common in RCC and increasing studies can pave the way to the clinical use of miRNAs as prognostic markers for metastasis. miR-10b, -139-5p, -130b and -199b-5p were associated with ccRCC metastasis and prognosis (98). In addition, the expression levels of miR-106b were significantly lower in tumors of patients who developed metastasis, with miR-106b being a potential predictive marker of early metastasis after nephrectomy in RCC patients (99).

In plasma, higher circulating expression levels of miR-221 were associated with poor overall survival in RCC patients (73). miR-187 was downregulated in the plasma and tumor tissue of ccRCC patients. Decreased miR-187 expression levels were associated with increased tumor grade and stage. Patients with high miR-187 expression survived 5 years, while of those with low miR-187 expression, only 42% survived (40).

In addition, miRNA-related SNPs may influence the recurrence and survival in RCC patients. Future investigations in larger populations and functional characterizations are necessary to validate these results (83).

Targeting therapy is one of the most effective approaches for the treatment of mRCC patients, however, the important issue is prediction of the response. One study showed that miR-141 was significantly downregulated in tumors of poor responders to sunitinib compared to good responders (100). Another study showed that miR-942 was the most accurate predictor of sunitinib efficacy for mRCC patients. A high expression of miR-942, -628-5p, -133a and -484 was significantly associated with decreased time to progression and overall survival in mRCC patients, and these miRNAs were also overexpressed in the sunitinib-resistant Caki-2 cell line in comparison with the sensitive cell line (85).

## 7. Therapy

With the significant roles that miRNAs play in the pathogenesis, increasing efforts are dedicated to the development of miRNA-based therapies. There is great interest in the potential application of the restoring functions of tumor suppressive miRNAs and the inhibiting oncogenic miRNAs.

For example, reintroducing miR-199a-3p in 769-P and Caki-1 RCC cell lines inhibited cell proliferation and caused G_1_-phase arrest (42). Restoration of miR-138 in RCC cells changed the EMT-like morphology and suppressed cell migration and invasion (31). Simultaneously expressed miR-424 and -381 synergistically inhibited proliferation, abrogated G2/M arrest, and induced apoptosis. The combination led to Cdc2 activation through WEE1 inhibition, which was more effective when cells were treated with the two miRNAs compared with either miRNA alone, indicating synergy between these miRNAs (101). miR-138 induced SN-12 cell senescence by downregulating EZH2 expression and upregulating P16 expression in ccRCC (33). In addition, the transient and stable overexpression of miR-205 in A498 cells resulted in the induction of G_0_/G_1_ cell-cycle arrest and apoptosis, decreased levels of cyclin D1 and c-Myc, suppressed cell proliferation, colony formation, migration, and invasion in RCC cells. miR-205 also inhibited tumor cell growth *in vivo* (44). Furthermore, miR-34a suppressed RCC cell growth, tube formation and metastasis *in vitro* and *in vivo* by targeting CD44 (102).

On the other hand, silencing of miR-210 expression decreased the viability of ACHN and Caki-2 cells and accumulation of Caki-2 in G_2_ phase of the cell cycle. Downregulation of miR-210 also reduced the migratory and invasive potential of ACHN metastatic RCC cells (103). The knockdown of miRNA-23b-3p expression in RCC cell lines caused an induction of apoptosis and reduced invasive abilities by inducing PTEN gene expression with a concomitant reduction in PI3-kinase, total Akt and IL-32 (59). In addition, the suppression of miR-155 inhibited cell proliferation and migratory activity and induced apoptosis in RCC cells by inhibiting BACH1 protein (61). Moreover, the downregulation of miR-7 with synthesized inhibitor suppressed cell migration *in vitro* as well as cell proliferation, and induced RCC cell apoptosis (54).

The miRNA expression can be controlled by epigenetic silencing, which is a regulatory mechanism of miRNA. Therefore, epigenetic modulation of the gene expression may be useful for modulating miRNA expression. In ccRCC cell lines, treatment with inhibitors of the DNA methyltransferase and histone deacetylase causes re-expression of silenced miRNAs with putative tumor suppressive function (104).

miRNA-based therapies may also be used together with other therapeutic strategies in pre-clinical studies. For instance, miR-381 increased sensitivity of 786-O cells to 5-FU by inhibiting WEE1 and increasing Cdc2 activity (105). In another study, miR-185 enhanced radiation-induced apoptosis and inhibition of proliferation by repressing the ATR pathway (106). Notably, the reintroduction of miR-141 *in vitro* led to EMT reversal and increased sensibility to a hypoxic environment (100).

## 8. Conclusion

Emerging evidence suggests that miRNAs have a significant impact on our understanding of the pathogenesis of RCC. More studies are required to accurately identify the mechanisms by which miRNAs affect RCC. Moreover, miRNAs present new potential tumor biomarkers that may improve our diagnostic, prognostic and predictive abilities and, consequently, cancer patient treatment strategy. Since the finding that miRNAs can have direct biological effects on cancer, there has been much interest in developing novel miRNA-based cancer therapies. The development of a useful miRNA therapy has the capability to revolutionize personalized cancer therapy.

## Figures and Tables

**Table I:** Downregulated microRNAs in renal cell carcinoma.

MicroRNAs	Specimen	Function	Target genes	Pathways/mechanisms involved	Refs.
miR-1/133a	RCC tissues, cell lines	Tumor suppressor	TAGLN2	Proliferation, invasion, apoptosis, cell cycle	23
miR-30c	RCC tissues, cell lines		Slug	Hypoxia, EMT	24
miR-30d	RCC tissues	Tumor suppressor	Cyclin E2	Proliferation, colony formation, cell cycle	25
miR-34a	RCC tissues	Tumor suppressor	c-Met, c-MYC, Notch1	Cell growth, cell cycle	26,27
miR-99a	RCC tissues		mTOR	Cells growth, clonality, migration, invasion, cell cycle	28
miR-133b	Cell lines	Tumor suppressor	MMP-9	Proliferation, migration, invasion	29
miR-135a	RCC tissues	Tumor suppressor	c-MYC	Cell proliferation, cell cycle	30
miR-138	Cell lines	Tumor suppressor	VIM, HIF-1α, EZH2	Migration, invasion, senescence	31–33
miR-141	RCC tissues		CDC25B	Cell growth, metastasis, EphA2/p-FAK/p-AKT/MMPs signaling	34,35
miR-143/145 cluster	RCC tissues		Hexokinase-2 (HK2)	Cell proliferation, invasion	36
miR-145	RCC tissues	Tumor suppressor	ADAM17, ANGPT2, NEDD9	Proliferation, migration	37,38
miR-182-5p	RCC tissues	Tumor suppressor	FLOT1	AKT/FOXO3a signaling	39
miR-187	Tumor tissue, plasma	Tumor suppressor	B7 homolog 3 (B7-H3)	Proliferation, tumor growth, motility	40
miR-192/194/215	Metastatic tumors		ZEB2, MDM2, TYMS	Migration, invasion, proliferation	41
miR-199a-3p	RCC tissues, cell lines		c-Met	HGF/c-Met signaling	42
miR-200c	RCC tissues		ZEB1	EMT, p-Akt, Akt	43
miR-205	RCC tissue, cell line		SFKs	Ras/Raf/ERK1/2 signaling, cell cycle, apoptosis, proliferation, colony formation, migration, invasion	44
miR-217	ccRCC tissues	Tumor suppressor		Proliferation, motility	45
miR-218	RCC tissues	Tumor suppressor	CAV2	Migration, invasion, focal adhesion	46
miR-508-3p/509-3p	RCC tissues, plasma	Tumor suppressor		Proliferation, apoptosis, migration	47
miR-509-5p	RCC tissues, plasma			Proliferation, migration, apoptosis	48
miR-584	Cell lines	Tumor suppressor	ROCK-1	Cell motility	49
miR-708	RCC tissues	Tumor suppressor	Survivin, ZEB2, BMI1	Cell growth, clonality, invasion, migration, apoptosis	50
miR-1285	RCC tissues	Tumor suppressor	TGM2	Proliferation, invasion, migration	51
miR-1291	RCC tissues	Tumor suppressor	SLC2A1/GLUT1	Cell proliferation, migration, invasion	52
miR-1826	RCC tissues	Tumor suppressor	β-catenin, MEK1	Proliferation, invasion, migration, apoptosis, cell cycle	53

EMT, epithelial to mesenchymal transition.

**Table II:** Upregulated microRNAs in renal cell carcinoma.

MicroRNAs	Specimen	Function	Target genes	Pathways/mechanisms involved	Refs.
miR-7	RCC tissues	Oncogene		Migration, proliferation, apoptosis	54
miR-21	RCC tissues, metastatic RCC, cell lines	Tumor marker	PDCD4, TPM1, PTEN, FASL, TIMP3	Growth, apoptosis, cell cycle, invasion, migration	55–58
miR-23b-3p	RCC tissues, cell lines		PTEN	Cell cycle, apoptosis, invasion, migration	59
miR-23b	Cell lines, RCC tissues	Oncogene	POX	HIF, apoptosis	60
miR-155	RCC tissues, cell lines	Oncogene	BACH1	Proliferation, migration, apoptosis	61
miR-210	Cell line		E2F3	Cell cycle, migration, invasion	62
miR-224/383	ccRCC tissues		DIO1	Tissue hypothyroidism	63
miR-590-5p	ACHN, 786-O cells	Oncomir	PBRM1	Proliferation, invasion, cell cycle	64

## References

[1] Siegel R, Ma J, Zou Z, Jemal A (2014). Cancer statistics, 2014. CA Cancer J Clin.

[2] Cohen HT, McGovern FJ (2005). Renal-cell carcinoma. N Engl J Med.

[3] Rini BI, Rathmell WK, Godley P (2008). Renal cell carcinoma. Curr Opin Oncol.

[4] Janzen NK, Kim HL, Figlin RA, Belldegrun AS (2003). Surveillance after radical or partial nephrectomy for localized renal cell carcinoma and management of recurrent disease. Urol Clin North Am.

[5] Bullock A, McDermott DF, Atkins MB (2010). Management of metastatic renal cell carcinoma in patients with poor prognosis. Cancer Manag Res.

[6] Lilleby W, Fossa SD (2005). Chemotherapy in metastatic renal cell cancer. World J Urol.

[7] Redova M, Svoboda M, Slaby O (2011). MicroRNAs and their target gene networks in renal cell carcinoma. Biochem Biophys Res Commun.

[8] Bartel DP (2009). MicroRNAs: target recognition and regulatory functions. Cell.

[9] Lee RC, Feinbaum RL, Ambros V (1993). The C. elegans heterochronic gene lin-4 encodes small RNAs with antisense complementarity to lin-14. Cell.

[10] Croce CM (2009). Causes and consequences of microRNA dysregulation in cancer. Nat Rev Genet.

[11] Rodriguez A, Griffiths-Jones S, Ashurst JL, Bradley A (2004). Identification of mammalian microRNA host genes and transcription units. Genome Res.

[12] Saini HK, Griffiths-Jones S, Enright AJ (2007). Genomic analysis of human microRNA transcripts. Proc Natl Acad Sci USA.

[13] Cai X, Hagedorn CH, Cullen BR (2004). Human microRNAs are processed from capped, polyadenylated transcripts that can also function as mRNAs. RNA.

[14] Lee Y, Kim M, Han J (2004). MicroRNA genes are transcribed by RNA polymerase II. EMBO J.

[15] Denli AM, Tops BB, Plasterk RH, Ketting RF, Hannon GJ (2004). Processing of primary microRNAs by the microprocessor complex. Nature.

[16] Han J, Lee Y, Yeom KH, Kim YK, Jin H, Kim VN (2004). The Drosha-DGCR8 complex in primary microRNA processing. Genes Dev.

[17] Lund E, Guttinger S, Calado A, Dahlberg JE, Kutay U (2004). Nuclear export of microRNA precursors. Science.

[18] Yi R, Qin Y, Macara IG, Cullen BR (2003). Exportin-5 mediates the nuclear export of pre-microRNAs and short hairpin RNAs. Genes Dev.

[19] Zhang B, Pan X, Cobb GP, Anderson TA (2007). microRNAs as oncogenes and tumor suppressors. Dev Biol.

[20] Jackson RJ, Standart N (2007). How do microRNAs regulate gene expression?. Sci STKE.

[21] Nilsen TW (2007). Mechanisms of microRNA-mediated gene regulation in animal cells. Trends Genet.

[22] Pillai RS, Bhattacharyya SN, Filipowicz W (2007). Repression of protein synthesis by miRNAs: how many mechanisms?. Trends Cell Biol.

[23] Kawakami K, Enokida H, Chiyomaru T (2012). The functional significance of miR-1 and miR-133a in renal cell carcinoma. Eur J Cancer.

[24] Huang J, Yao X, Zhang J (2013). Hypoxia-induced downregulation of miR-30c promotes epithelial-mesenchymal transition in human renal cell carcinoma. Cancer Sci.

[25] Yu H, Lin X, Wang F (2014). Proliferation inhibition and the underlying molecular mechanisms of microRNA-30d in renal carcinoma cells. Oncol Lett.

[26] Yamamura S, Saini S, Majid S (2012). MicroRNA-34a suppresses malignant transformation by targeting c-Myc transcriptional complexes in human renal cell carcinoma. Carcinogenesis.

[27] Zhang C, Mo R, Yin B, Zhou L, Liu Y, Fan J (2014). Tumor suppressor microRNA-34a inhibits cell proliferation by targeting Notch1 in renal cell carcinoma. Oncol Lett.

[28] Cui L, Zhou H, Zhao H (2012). MicroRNA-99a induces G1-phase cell cycle arrest and suppresses tumorigenicity in renal cell carcinoma. BMC Cancer.

[29] Wu D, Pan H, Zhou Y, Zhou J, Fan Y, Qu P (2014). microRNA-133b downregulation and inhibition of cell proliferation, migration and invasion by targeting matrix metallopeptidase-9 in renal cell carcinoma. Mol Med Rep.

[30] Yamada Y, Hidaka H, Seki N (2013). Tumor-suppressive microRNA-135a inhibits cancer cell proliferation by targeting the c-MYC oncogene in renal cell carcinoma. Cancer Sci.

[31] Yamasaki T, Seki N, Yamada Y (2012). Tumor suppressive microRNA138 contributes to cell migration and invasion through its targeting of vimentin in renal cell carcinoma. Int J Oncol.

[32] Song T, Zhang X, Wang C (2011). MiR-138 suppresses expression of hypoxia-inducible factor 1α (HIF-1α) in clear cell renal cell carcinoma 786-O cells. Asian Pac J Cancer Prev.

[33] Liang J, Zhang Y, Jiang G (2013). MiR-138 induces renal carcinoma cell senescence by targeting EZH2 and is down-regulated in human clear cell renal cell carcinoma. Oncol Res.

[34] Yu XY, Zhang Z, Liu J, Zhan B, Kong CZ (2013). MicroRNA-141 is downregulated in human renal cell carcinoma and regulates cell survival by targeting CDC25B. Onco Targets Ther.

[35] Chen X, Wang X, Ruan A (2014). miR-141 is a key regulator of renal cell carcinoma proliferation and metastasis by controlling EphA2 expression. Clin Cancer Res.

[36] Yoshino H, Enokida H, Itesako T (2013). Tumor-suppressive microRNA-143/145 cluster targets hexokinase-2 in renal cell carcinoma. Cancer Sci.

[37] Doberstein K, Steinmeyer N, Hartmetz AK (2013). MicroRNA-145 targets the metalloprotease ADAM17 and is suppressed in renal cell carcinoma patients. Neoplasia.

[38] Lu R, Ji Z, Li X (2014). miR-145 functions as tumor suppressor and targets two oncogenes, ANGPT2 and NEDD9, in renal cell carcinoma. J Cancer Res Clin Oncol.

[39] Xu X, Wu J, Li S (2014). Downregulation of microRNA-182-5p contributes to renal cell carcinoma proliferation via activating the AKT/FOXO3a signaling pathway. Mol Cancer.

[40] Zhao J, Lei T, Xu C (2013). MicroRNA-187, down-regulated in clear cell renal cell carcinoma and associated with lower survival, inhibits cell growth and migration though targeting B7-H3. Biochem Biophys Res Commun.

[41] Khella HW, Bakhet M, Allo G (2013). miR-192, miR-194 and miR-215: a convergent microRNA network suppressing tumor progression in renal cell carcinoma. Carcinogenesis.

[42] Huang J, Dong B, Zhang J (2014). miR-199a-3p inhibits hepatocyte growth factor/c-Met signaling in renal cancer carcinoma. Tumour Biol.

[43] Wang X, Chen X, Wang R (2013). microRNA-200c modulates the epithelial-to-mesenchymal transition in human renal cell carcinoma metastasis. Oncol Rep.

[44] Majid S, Saini S, Dar AA (2011). MicroRNA-205 inhibits Src-mediated oncogenic pathways in renal cancer. Cancer Res.

[45] Li H, Zhao J, Zhang JW (2013). MicroRNA-217, down-regulated in clear cell renal cell carcinoma and associated with lower survival, suppresses cell proliferation and migration. Neoplasma.

[46] Yamasaki T, Seki N, Yoshino H (2013). MicroRNA-218 inhibits cell migration and invasion in renal cell carcinoma through targeting caveolin-2 involved in focal adhesion pathway. J Urol.

[47] Zhai Q, Zhou L, Zhao C (2012). Identification of miR-508-3p and miR-509-3p that are associated with cell invasion and migration and involved in the apoptosis of renal cell carcinoma. Biochem Biophys Res Commun.

[48] Zhang WB, Pan ZQ, Yang QS, Zheng XM (2013). Tumor suppressive miR-509-5p contributes to cell migration, proliferation and antiapoptosis in renal cell carcinoma. Ir J Med Sci.

[49] Ueno K, Hirata H, Shahryari V (2011). Tumour suppressor microRNA-584 directly targets oncogene Rock-1 and decreases invasion ability in human clear cell renal cell carcinoma. Br J Cancer.

[50] Saini S, Yamamura S, Majid S (2011). MicroRNA-708 induces apoptosis and suppresses tumorigenicity in renal cancer cells. Cancer Res.

[51] Hidaka H, Seki N, Yoshino H (2012). Tumor suppressive microRNA-1285 regulates novel molecular targets: aberrant expression and functional significance in renal cell carcinoma. Oncotarget.

[52] Yamasaki T, Seki N, Yoshino H (2013). Tumor-suppressive microRNA-1291 directly regulates glucose transporter 1 in renal cell carcinoma. Cancer Sci.

[53] Hirata H, Hinoda Y, Ueno K, Nakajima K, Ishii N, Dahiya R (2012). MicroRNA-1826 directly targets beta-catenin (CTNNB1) and MEK1 (MAP2K1) in VHL-inactivated renal cancer. Carcinogenesis.

[54] Yu Z, Ni L, Chen D (2013). Identification of miR-7 as an oncogene in renal cell carcinoma. J Mol Histol.

[55] Li X, Xin S, He Z (2014). MicroRNA-21 (miR-21) post-transcriptionally downregulates tumor suppressor PDCD4 and promotes cell transformation, proliferation, and metastasis in renal cell carcinoma. Cell Physiol Biochem.

[56] Lv L, Huang F, Mao H (2013). MicroRNA-21 is overexpressed in renal cell carcinoma. Int J Biol Markers.

[57] Dey N, Das F, Ghosh-Choudhury N (2012). microRNA-21 governs TORC1 activation in renal cancer cell proliferation and invasion. PLoS One.

[58] Zhang A, Liu Y, Shen Y, Xu Y, Li X (2011). miR-21 modulates cell apoptosis by targeting multiple genes in renal cell carcinoma. Urology.

[59] Zaman MS, Thamminana S, Shahryari V (2012). Inhibition of PTEN gene expression by oncogenic miR-23b-3p in renal cancer. PLoS One.

[60] Liu W, Zabirnyk O, Wang H (2010). miR-23b targets proline oxidase, a novel tumor suppressor protein in renal cancer. Oncogene.

[61] Li S, Chen T, Zhong Z, Wang Y, Li Y, Zhao X (2012). microRNA-155 silencing inhibits proliferation and migration and induces apoptosis by upregulating BACH1 in renal cancer cells. Mol Med Rep.

[62] Nakada C, Tsukamoto Y, Matsuura K (2011). Overexpression of miR-210, a downstream target of HIF1alpha, causes centrosome amplification in renal carcinoma cells. J Pathol.

[63] Boguslawska J, Wojcicka A, Piekielko-Witkowska A, Master A, Nauman A (2011). MiR-224 targets the 3′UTR of type 1 5′-iodothy-ronine deiodinase possibly contributing to tissue hypothyroidism in renal cancer. PLoS One.

[64] Xiao X, Tang C, Xiao S, Fu C, Yu P (2013). Enhancement of proliferation and invasion by MicroRNA-590-5p via targeting PBRM1 in clear cell renal carcinoma cells. Oncol Res.

[65] Jung M, Mollenkopf HJ, Grimm C (2009). MicroRNA profiling of clear cell renal cell cancer identifies a robust signature to define renal malignancy. J Cell Mol Med.

[66] Gottardo F, Liu CG, Ferracin M (2007). Micro-RNA profiling in kidney and bladder cancers. Urol Oncol.

[67] Cheng T, Wang L, Li Y, Huang C, Zeng L, Yang J (2013). Differential microRNA expression in renal cell carcinoma. Oncol Lett.

[68] Faragalla H, Youssef YM, Scorilas A (2012). The clinical utility of miR-21 as a diagnostic and prognostic marker for renal cell carcinoma. J Mol Diagn.

[69] Silva-Santos RM, Costa-Pinheiro P, Luis A (2013). MicroRNA profile: a promising ancillary tool for accurate renal cell tumour diagnosis. Br J Cancer.

[70] Wotschofsky Z, Liep J, Meyer HA (2012). Identification of metastamirs as metastasis-associated microRNAs in clear cell renal cell carcinomas. Int J Biol Sci.

[71] Heinzelmann J, Henning B, Sanjmyatav J (2011). Specific miRNA signatures are associated with metastasis and poor prognosis in clear cell renal cell carcinoma. World J Urol.

[72] Redova M, Poprach A, Nekvindova J (2012). Circulating miR-378 and miR-451 in serum are potential biomarkers for renal cell carcinoma. J Transl Med.

[73] Teixeira AL, Ferreira M, Silva J (2014). Higher circulating expression levels of miR-221 associated with poor overall survival in renal cell carcinoma patients. Tumour Biol.

[74] Lichner Z, Mejia-Guerrero S, Ignacak M (2012). Pleiotropic action of renal cell carcinoma-dysregulated miRNAs on hypoxia-related signaling pathways. Am J Pathol.

[75] Neal CS, Michael MZ, Rawlings LH, Van der Hoek MB, Gleadle JM (2010). The VHL-dependent regulation of microRNAs in renal cancer. BMC Med.

[76] Lian JH, Wang WH, Wang JQ, Zhang YH, Li Y (2013). MicroRNA-122 promotes proliferation, invasion and migration of renal cell carcinoma cells through the PI3K/Akt signaling pathway. Asian Pac J Cancer Prev.

[77] Bera A, Ghosh-Choudhury N, Dey N (2013). NFkappaB-mediated cyclin D1 expression by microRNA-21 influences renal cancer cell proliferation. Cell Signal.

[78] Bera A, Das F, Ghosh-Choudhury N, Kasinath BS, Abboud HE, Choudhury GG (2014). microRNA-21-induced dissociation of PDCD4 from rictor contributes to Akt-IKKbeta-mTORC1 axis to regulate renal cancer cell invasion. Exp Cell Res.

[79] Hildebrandt MA, Gu J, Lin J (2010). Hsa-miR-9 methylation status is associated with cancer development and metastatic recurrence in patients with clear cell renal cell carcinoma. Oncogene.

[80] Gebauer K, Peters I, Dubrowinskaja N (2013). Hsa-mir-124-3 CpG island methylation is associated with advanced tumours and disease recurrence of patients with clear cell renal cell carcinoma. Br J Cancer.

[81] Beresneva EV, Rykov SV, Hodyrev DS (2013). Methylation profile of group of miRNA genes in clear cell renal cell carcinoma; involvement in cancer progression. Genetika.

[82] Creighton CJ, Morgan M, Gunaratne PH (2013). Comprehensive molecular characterization of clear cell renal cell carcinoma. Nature.

[83] Lin J, Horikawa Y, Tamboli P, Clague J, Wood CG, Wu X (2010). Genetic variations in microRNA-related genes are associated with survival and recurrence in patients with renal cell carcinoma. Carcinogenesis.

[84] Hell MP, Thoma CR, Fankhauser N, Christinat Y, Weber TC, Krek W (2014). miR-28-5p promotes chromosomal instability in VHL-associated cancers by inhibiting Mad2 translation. Cancer Res.

[85] Prior C, Perez-Gracia JL, Garcia-Donas J (2014). Identification of tissue microRNAs predictive of sunitinib activity in patients with metastatic renal cell carcinoma. PLoS One.

[86] Powers MP, Alvarez K, Kim HJ, Monzon FA (2011). Molecular classification of adult renal epithelial neoplasms using microRNA expression and virtual karyotyping. Diagn Mol Pathol.

[87] Zaravinos A, Lambrou GI, Mourmouras N (2014). New miRNA profiles accurately distinguish renal cell carcinomas and upper tract urothelial carcinomas from the normal kidney. PLoS One.

[88] Youssef YM, White NM, Grigull J (2011). Accurate molecular classification of kidney cancer subtypes using microRNA signature. Eur Urol.

[89] Wulfken LM, Moritz R, Ohlmann C (2011). MicroRNAs in renal cell carcinoma: diagnostic implications of serum miR-1233 levels. PLoS One.

[90] Iwamoto H, Kanda Y, Sejima T, Osaki M, Okada F, Takenaka A (2014). Serum miR-210 as a potential biomarker of early clear cell renal cell carcinoma. Int J Oncol.

[91] Zhao A, Li G, Peoc’h M, Genin C, Gigante M (2013). Serum miR-210 as a novel biomarker for molecular diagnosis of clear cell renal cell carcinoma. Exp Mol Pathol.

[92] Von Brandenstein M, Pandarakalam JJ, Kroon L (2012). MicroRNA 15a, inversely correlated to PKCalpha, is a potential marker to differentiate between benign and malignant renal tumors in biopsy and urine samples. Am J Pathol.

[93] Gao Y, Zhao H, Lu Y, Li H, Yan G (2014). MicroRNAs as potential diagnostic biomarkers in renal cell carcinoma. Tumour Biol.

[94] Shinmei S, Sakamoto N, Goto K (2013). MicroRNA-155 is a predictive marker for survival in patients with clear cell renal cell carcinoma. Int J Urol.

[95] Slaby O, Redova M, Poprach A (2012). Identification of MicroRNAs associated with early relapse after nephrectomy in renal cell carcinoma patients. Genes Chromosomes Cancer.

[96] Wang G, Chen L, Meng J, Chen M, Zhuang L, Zhang L (2013). Overexpression of microRNA-100 predicts an unfavorable prognosis in renal cell carcinoma. Int Urol Nephrol.

[97] Zhao JJ, Chen PJ, Duan RQ, Li KJ, Wang YZ, Li Y (2014). Up-regulation of miR-630 in clear cell renal cell carcinoma is associated with lower overall survival. Int J Clin Exp Pathol.

[98] Wu X, Weng L, Li X (2012). Identification of a 4-microRNA signature for clear cell renal cell carcinoma metastasis and prognosis. PLoS One.

[99] Slaby O, Jancovicova J, Lakomy R (2010). Expression of miRNA-106b in conventional renal cell carcinoma is a potential marker for prediction of early metastasis after nephrectomy. J Exp Clin Cancer Res.

[100] Berkers J, Govaere O, Wolter P (2013). A possible role for microRNA-141 down-regulation in sunitinib resistant metastatic clear cell renal cell carcinoma through induction of epithelial-to-mesenchymal transition and hypoxia resistance. J Urol.

[101] Chen B, Duan L, Yin G, Tan J, Jiang X (2013). Simultaneously expressed miR-424 and miR-381 synergistically suppress the proliferation and survival of renal cancer cells - Cdc2 activity is up-regulated by targeting WEE1. Clinics.

[102] Yu G, Li H, Wang J (2014). miRNA-34a suppresses cell proliferation and metastasis by targeting CD44 in human renal carcinoma cells. J Urol.

[103] Redova M, Poprach A, Besse A (2013). MiR-210 expression in tumor tissue and in vitro effects of its silencing in renal cell carcinoma. Tumour Biol.

[104] Schiffgen M, Schmidt DH, von Rucker A, Muller SC, Ellinger J (2013). Epigenetic regulation of microRNA expression in renal cell carcinoma. Biochem Biophys Res Commun.

[105] Chen B, Duan L, Yin G, Tan J, Jiang X (2013). miR-381, a novel intrinsic WEE1 inhibitor, sensitizes renal cancer cells to 5-FU by up-regulation of Cdc2 activities in 786-O. J Chemother.

[106] Wang J, He J, Su F (2013). Repression of ATR pathway by miR-185 enhances radiation-induced apoptosis and proliferation inhibition. Cell Death Dis.

